# Supplementary material for: Improving tolerance to fluctuating light through adaptive laboratory evolution in the cyanobacterium Synechocystis
Source: Nat Commun. 2026 May 4;17:4025. doi: 10.1038/s41467-026-72689-x (PMC13139465; doi:10.1038/s41467-026-72689-x)
Supplement: Supplementary file 1 — Supplementary Information [file 41467_2026_72689_MOESM1_ESM.pdf]

SUPPLEMENTARY INFORMATION

Improving tolerance to fluctuating light through adaptive laboratory evolution in the cyanobacterium *Synechocystis*

Theo Figueroa-Gonzalez, Weiyang Chen, Eslam M. Abdel-Salam, Daniel Štipl, Josef Komenda, Milena Zhivkovikj, Marcel Dann & Dario Leister

Supplementary Figures

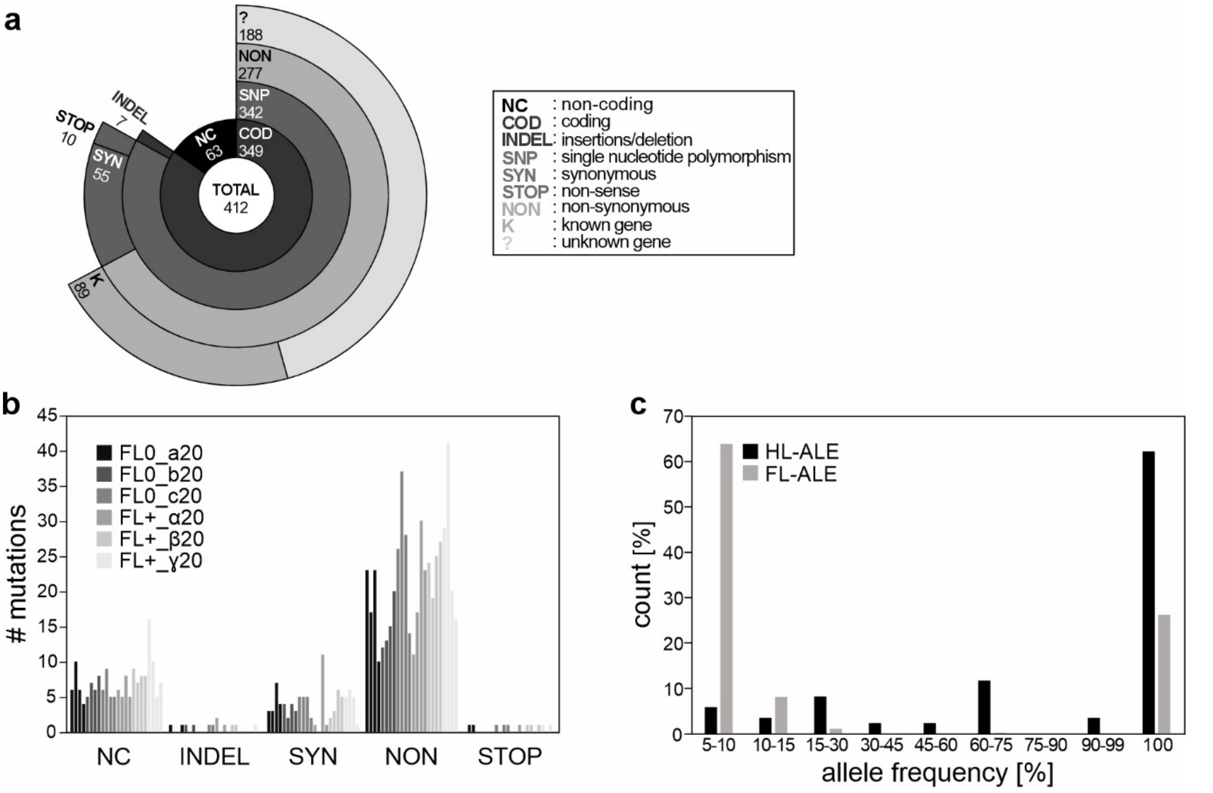

Supplementary Fig. 1 l Analysis of the mutational landscape observed in FL-adapted strains.

a, A hierarchical sunburst plot illustrates the distribution of novel alleles (absent in LT and WT strains) across four levels of classification: 1. Genomic context: Coding (COD) vs. Non-coding (NC) regions. 2. Mutation type in coding regions: Single-nucleotide polymorphisms (SNP) vs. Insertion/Deletions (INDELs). 3. SNP effect: Non-synonymous (NON), Synonymous (SYN) or Premature stop codon (STOP). 4. Functional annotation of non-synonymous mutations: Known/characterised (K) vs. Unknown/uncharacterised (?) genes.

b, Bar chart quantifying the relative abundance of mutations across five categories: NC, INDEL, NON, SYN, and STOP. Underlying data is provided in **Source Data 2.**

c, The frequency distribution of the total mutations observed in the two ALE experiments is presented, categorized into nine bins. This distribution is compared to previously reported data from high light (HL) ALE experiments conducted without external mutagens (see main text).

Underlying data is provided in **Source Data 3.**

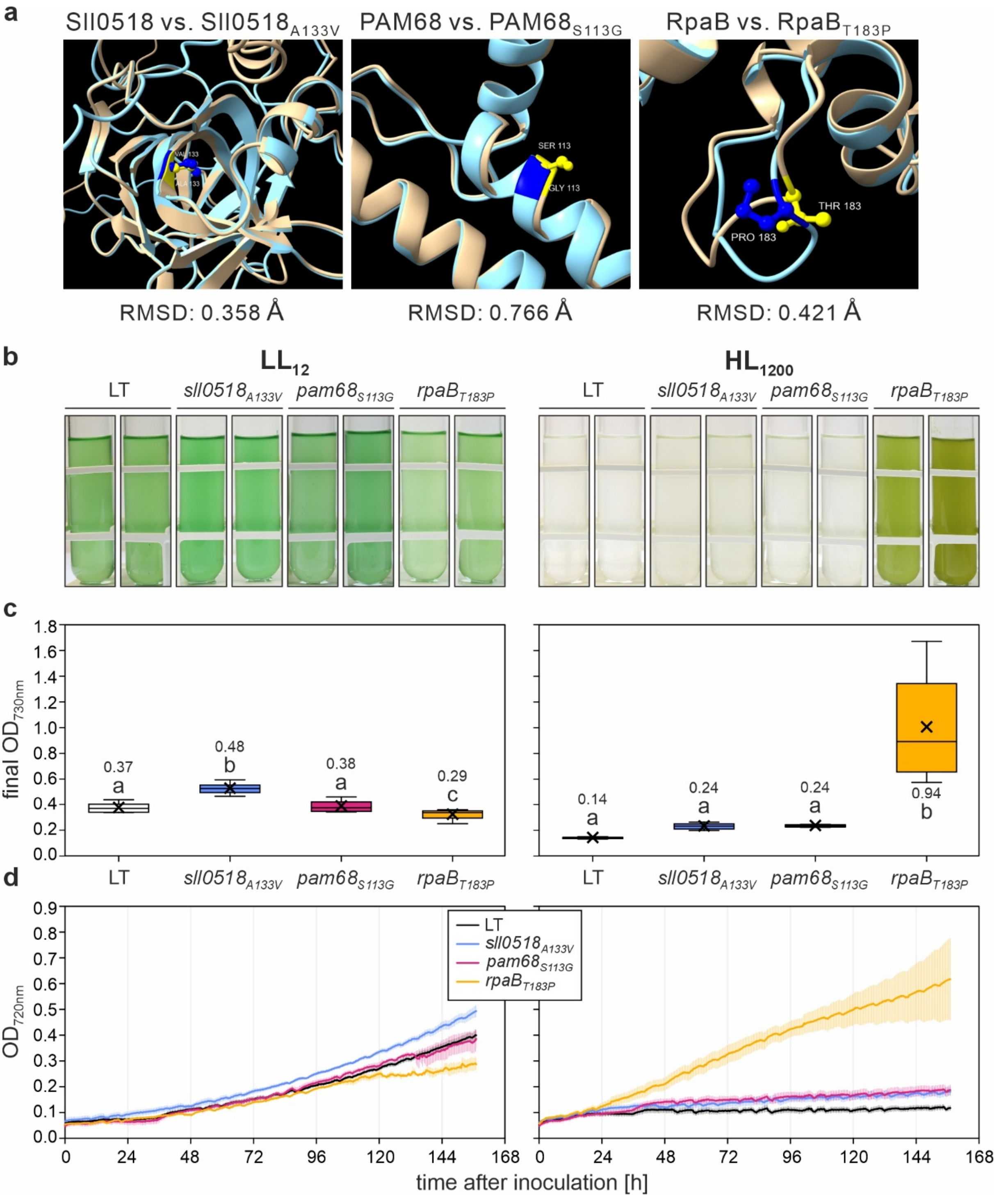

Supplementary Fig. 2 l Structural predictions of Sll0518$_{A133V}$, Pam68$_{S113G}$, and RpaB$_{T183P}$, and growth characteristics of their mutants and the LT strain under continuous LL$_{12}$ and HL$_{1200}$ conditions.

a, Close-up of the structural alignments of Sll0518/Sll0518$_{A133V}$, Pam68/Pam68$_{S113G}$, and RpaB/RpaB$_{T183P}$, as predicted by AlphaFold3. The full predicted structures are provided in **Source Data 4**. RMSD (root mean square deviation) values below 1.0 Å indicate no significant changes in the 3D structure compared to the wild-type protein reference.

b, Visual representation of liquid cultures for the four strains cultivated under LL (left) or HL (right) conditions at 23 °C with 100 mL min^{-1} aeration, photographed seven days after post-inoculation. Data were collected from cultures grown in multi-cultivators.

c, Quantitative analysis of cell density (OD_{730nm}) for the four strains grown under conditions described in b. Box plots are presented, with lowercase letters denoting statistically significant differences ($p \leq 0.05$) as determined by post-hoc Bonferroni-Holm simultaneous comparison of all measurements (n = 8 biological replicates) following significant between-group differences detected by one-factorial ANOVA.

d, Growth kinetics of the four strains under LL and HL conditions, monitored automatically by the multi-cultivators measuring OD_{720nm}. The solid lines show the mean, and error bars represent the standard deviation (n = 4 biological replicates).

Statistical data in panel c are presented as box plots, showing individual data points, median (horizontal lines), mean (crosses), interquartile range (box), and 1.5× interquartile range (whiskers). Raw data are provided in **Source Data 4.**

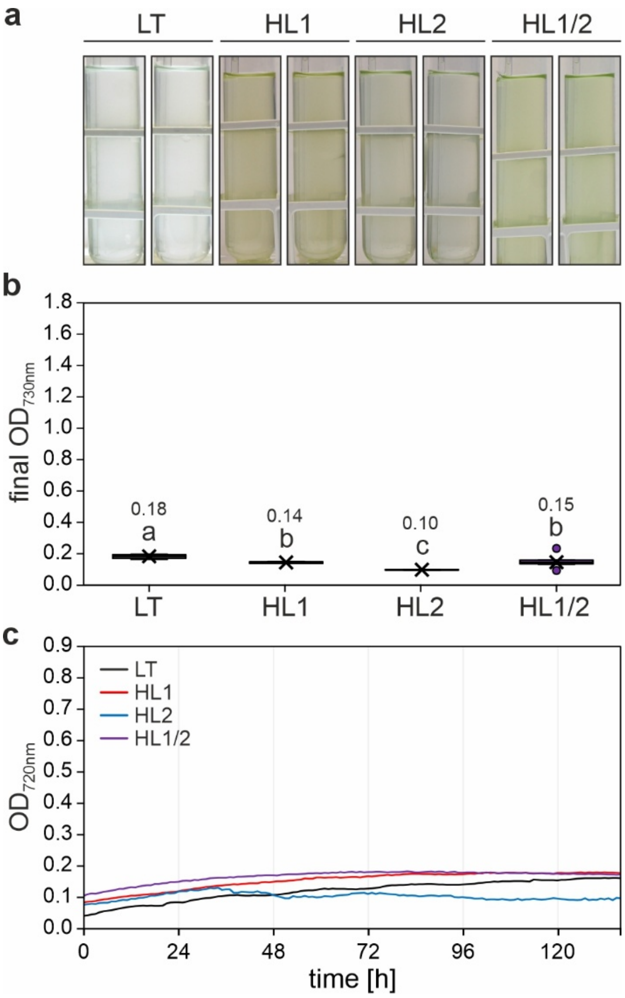

Supplementary Fig. 3 l Growth characteristics of HL-tolerant strains HL1, HL2, HL1/2 and the LT strain under FL+$_{\mathbf{final}}$ conditions.

a, Visual representation of liquid cultures for the four strains cultivated in multi-cultivators under FL+$_{final}$ conditions at 23 °C with 100 mL min^{-1} aeration, photographed seven days post-inoculation.

b, Quantitative analysis of cell density (OD_{730nm}) for the four strains grown under conditions as in a. Box plots are presented, with lowercase letters denoting statistically significant differences ($p \leq 0.05$) as determined by post-hoc Bonferroni-Holm simultaneous comparison of all measurements (n = 8 biological replicates) following significant between-group differences detected by one-factorial ANOVA.

c, Growth kinetics of the four strains under final FL+ conditions, monitored automatically by multi-cultivators measuring OD_{720nm}. The solid lines show the mean, and error bars represent the standard deviation (*n* = 4 biological replicates).

Statistical data in panel b are presented as box plots, showing individual data points, median (horizontal lines), mean (crosses), interquartile range (box), and 1.5× interquartile range (whiskers). Raw data are provided in **Source Data 4.**

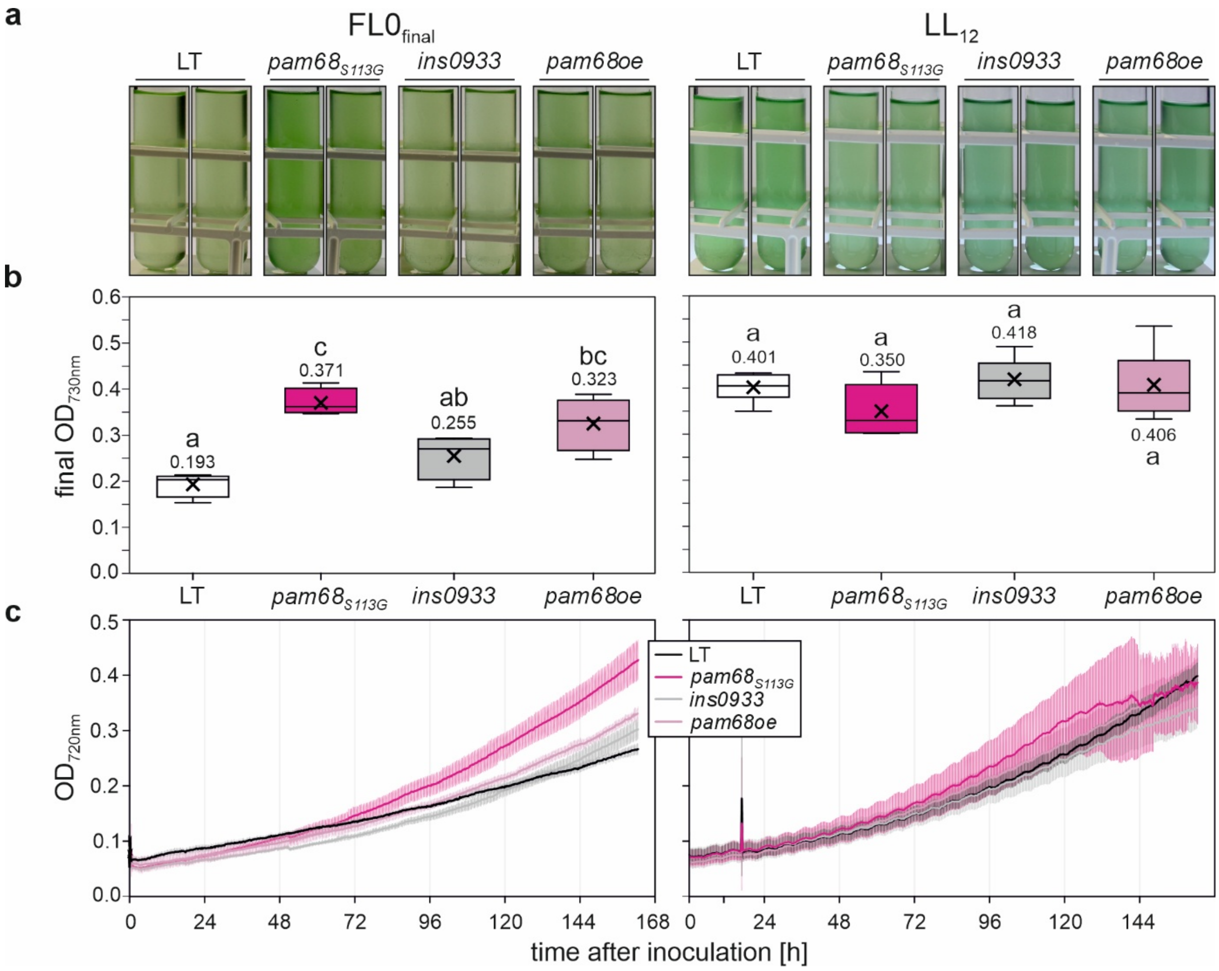

Supplementary Fig. 4 | Growth characteristics of the *$pam68_{S113G}$*, *ins0933*, *pam68oe* and the LT strain under two different light conditions.

a, Visual representation of liquid cultures of the LT, *ins0933*, *pam68oe* and *$pam68_{S113G}$* strains cultivated in multi-cultivators under $FL0_{final}$ and constant LL_{12} conditions at 23 °C with 100 mL min^{-1} aeration, photographed seven days post-inoculation.

b, Quantitative analysis of cell density (OD_{730nm}) for the four strains grown under the conditions as in a. Box plots are presented, with lowercase letters denoting statistically significant differences ($p \leq 0.05$) as determined by *post-hoc* Bonferroni-Holm simultaneous comparison of all measurements (*n* = 4 biological replicates for FL0 condition and *n* = 6 biological replicates for LL_{12} condition, respectively) following significant between-group differences detected by one-factorial ANOVA.

c, Growth kinetics of the four strains, monitored automatically by multi-cultivators measuring OD_{720nm}. The solid lines show the mean, and error bars represent the standard deviation (*n* = 4 biological replicates for $FL0_{final}$ condition and *n* = 6 biological replicates for LL_{12} condition, respectively).

Statistical data in panel **b** are presented as box plots, showing individual data points, median (horizontal lines), mean (crosses), interquartile range (box), and 1.5× interquartile range (whiskers). Raw data are provided in **Source Data 5.**

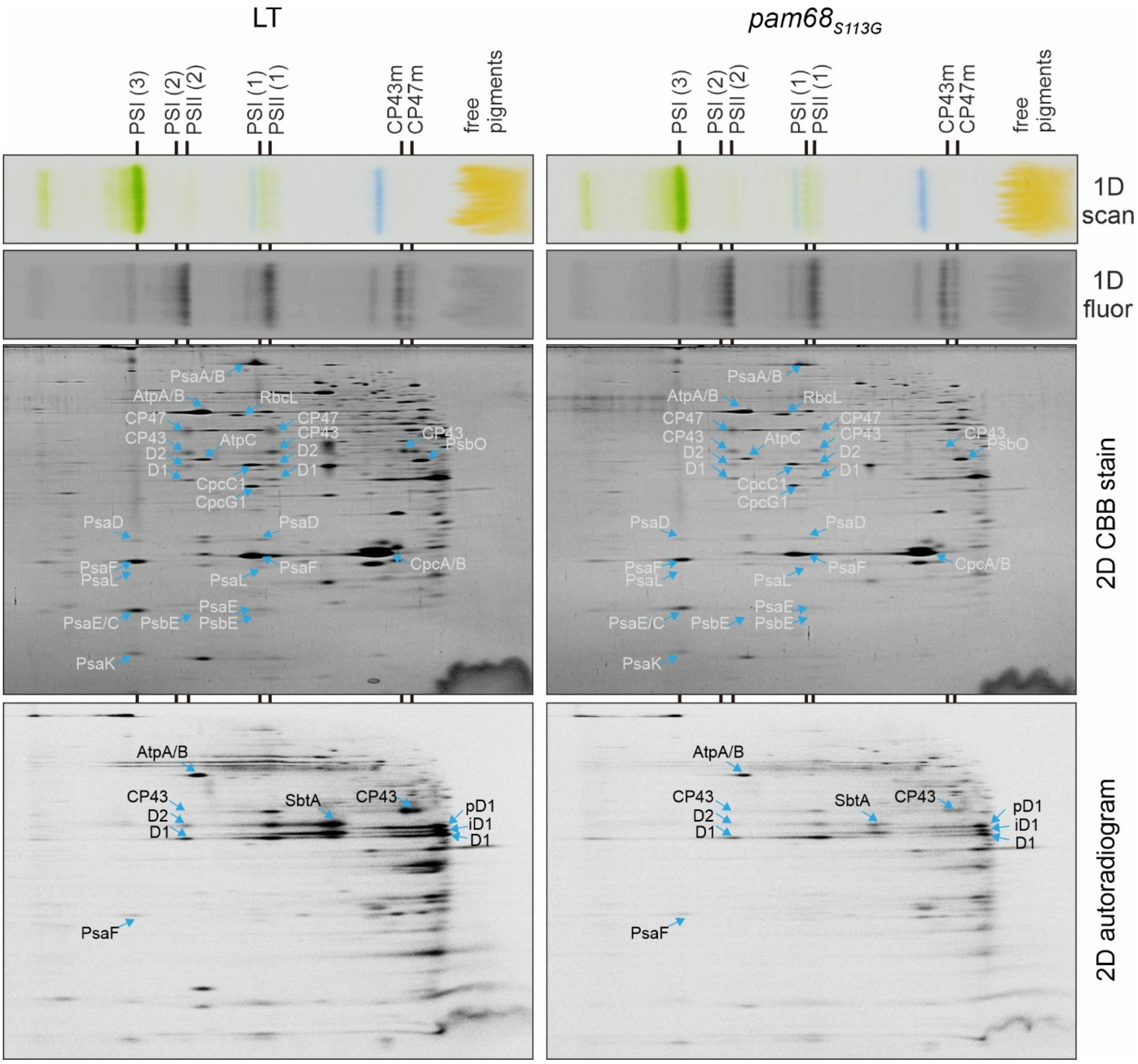

Supplementary Fig. 5 l *De-novo* synthesis of PSII proteins in the *pam68*$_{S113G}$ mutant. Cells were radiolabelled with a mixture of [^{35}S]Met/Cys for 20 min at 500 µmol photons m^{-2} s^{-1}. Membrane proteins isolated from the labelled cells were separated by 2D-CN/SDS-PAGE. The 1D native gels were photographed (1D scan) and examined for Chl fluorescence (1D fluor). After 2D SDS-PAGE, the gels were stained with Coomassie Brilliant Blue (2D CBB stain), dried, and imaged by a phosphoimager to detect the labelled proteins (2D autoradiogram). PSI (3): PSI trimer; PSI (2): PSI dimer; PSII (2): PSII dimer; PSI (1): PSI monomer; PSII (1): PSII monomer; CP43m: CP43 module; CP47m: CP47 module; pD1: D1 precursor; iD1: incompletely processed form of the D1 precursor.

Experiments were repeated independently two times with similar results, and representative images of one biological replicate are shown. Raw data are provided in **Source Data 5.**

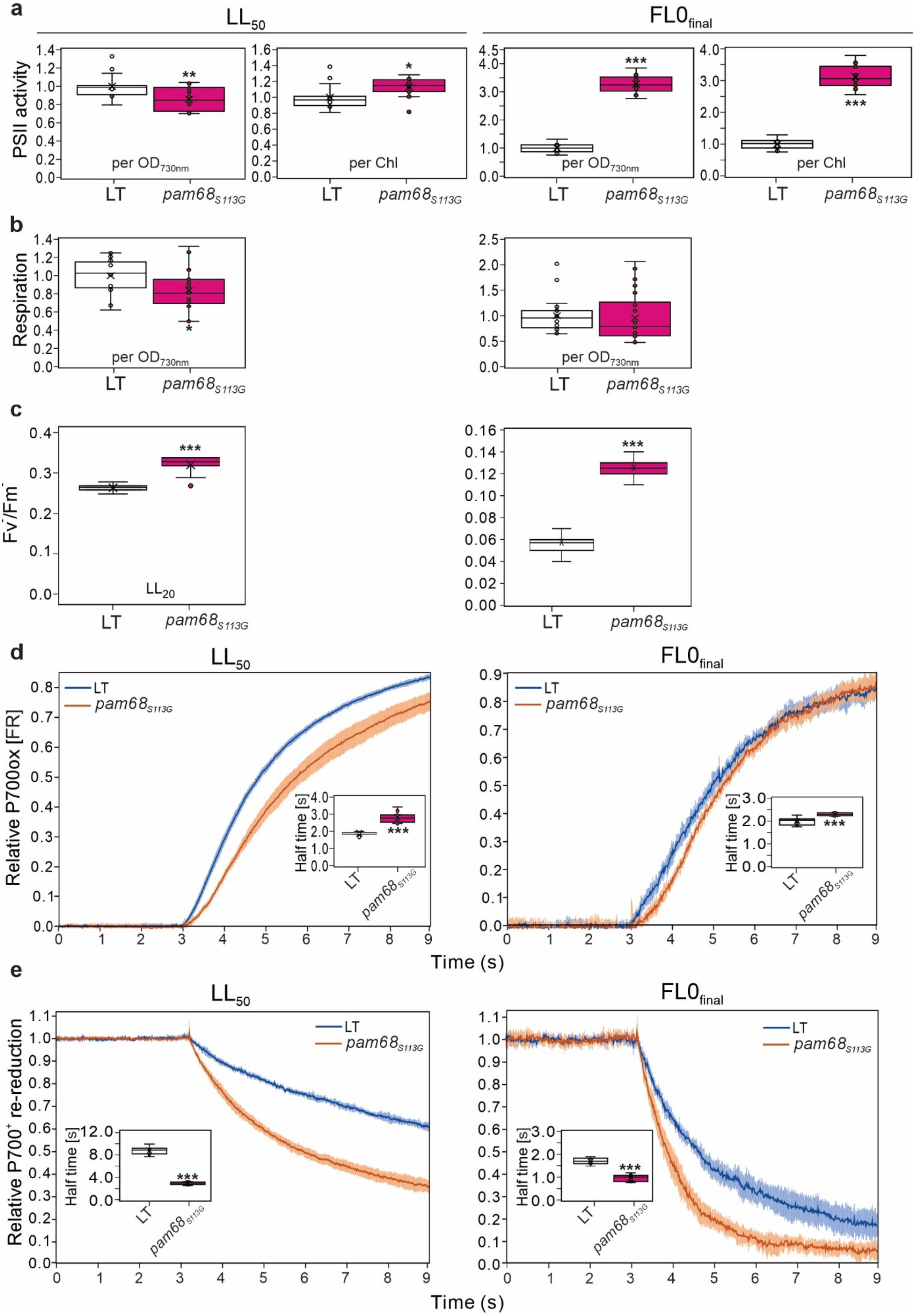

Supplementary Fig. 6 | Physiological measurements of *pam68*$_{S113G}$.

a, PSII activity. PSII activity was determined by measuring steady-state oxygen evolution in the presence of 0.5 mM DCBQ and 1 mM $K_3Fe(CN)_6$ under saturating light conditions. Measurements were performed on undiluted samples grown for 7 days at LL_{50} and $FL0_{final}$ (*n*=16 biological replicates for LL_{50}, and *n*=17 for $FL0_{final}$). Data were normalized to OD_{730} =1 and Chl a content. Mean activities for LT and *pam68*$_{S113G}$ cells were 0.90±0.14 and 0.78±0.19 µmol O_2/mL/h (LL_{50}), and 0.16±0.02 and 0.52±0.05 µmol O_2/mL/h ($FL0_{final}$), respectively. Normalized to Chl content, values were 176±6 and 199±21 µmol O_2/mg Chl/h (LL_{50}), and 47±7 and 145 ± 14 µmol O_2/mg Chl/h ($FL0_{final}$). Relative PSII activity was normalized to averages and presented as box plots ($p=3.36x10^{-3}$ and $1.05x10^{-2}$ for LL_{50}; $1.22x10^{-23}$ and $5.11x10^{-22}$ for $FL0_{final}$.

b, Cellular respiration. Respiration rates were measured as oxygen consumption in the dark over 5 min (*n*=17 biological replicates for LL_{50}, and *n*=22 for $FL0_{final}$). Mean values for LT and *pam68*$_{S113G}$ were 0.074±0.024 and 0.064±0.030 µmol O_2/mL/h (LL_{50}), and 0.143±0.052 and 0.097±0.018 µmol O_2/mL/h ($FL0_{final}$). Statistical significance: $p=2.70x10^{-2}$ (LL_{50}) and $7.21x10^{-1}$ ($FL0_{final}$).

c, Chlorophyll fluorescence. Fluorescence was measured in dark-acclimated, OD_{730}-normalised cells under increasing red-orange light (*n*=25 biological replicates for LL_{50}, *n*=7 for $FL0_{final}$.). The apparent PSII quantum yield (F_v^-/F_m^-) was calculated using saturating pulses (~1200 µmol photons m^{-2} s^{-1}). Significant differences were observed ($p=4.55x10^{-16}$ for LL_{50}; $2.75x10^{-21}$ for $FL0_{final}$).

d, e, P700 kinetics. P700 oxidation (**d**) and re-reduction (**e**) were monitored after dark incubation (≥16 h) using FR light and dark incubation to follow P700 oxidation and re-reduction, respectively (see **Methods**). Data represent averages from 12 (LL_{50}) and 8 ($FL0_{final}$) biological replicates. Insets show half-times of oxidation and re-reduction. Significant differences were observed (oxidation: $p=6.30x10^{-10}$ (LL_{50}), $5.20x10^{-4}$ ($FL0_{final}$); re-reduction: $p=2.33x10^{-12}$ (LL_{50}), $2.46x10^{-7}$ ($FL0_{final}$).

Statistical significance was assessed using two-sided Student's t-Test (*p≤0.05, p**≤0.01, p***≤0.001.

Raw data are provided in **Source Data 5**.

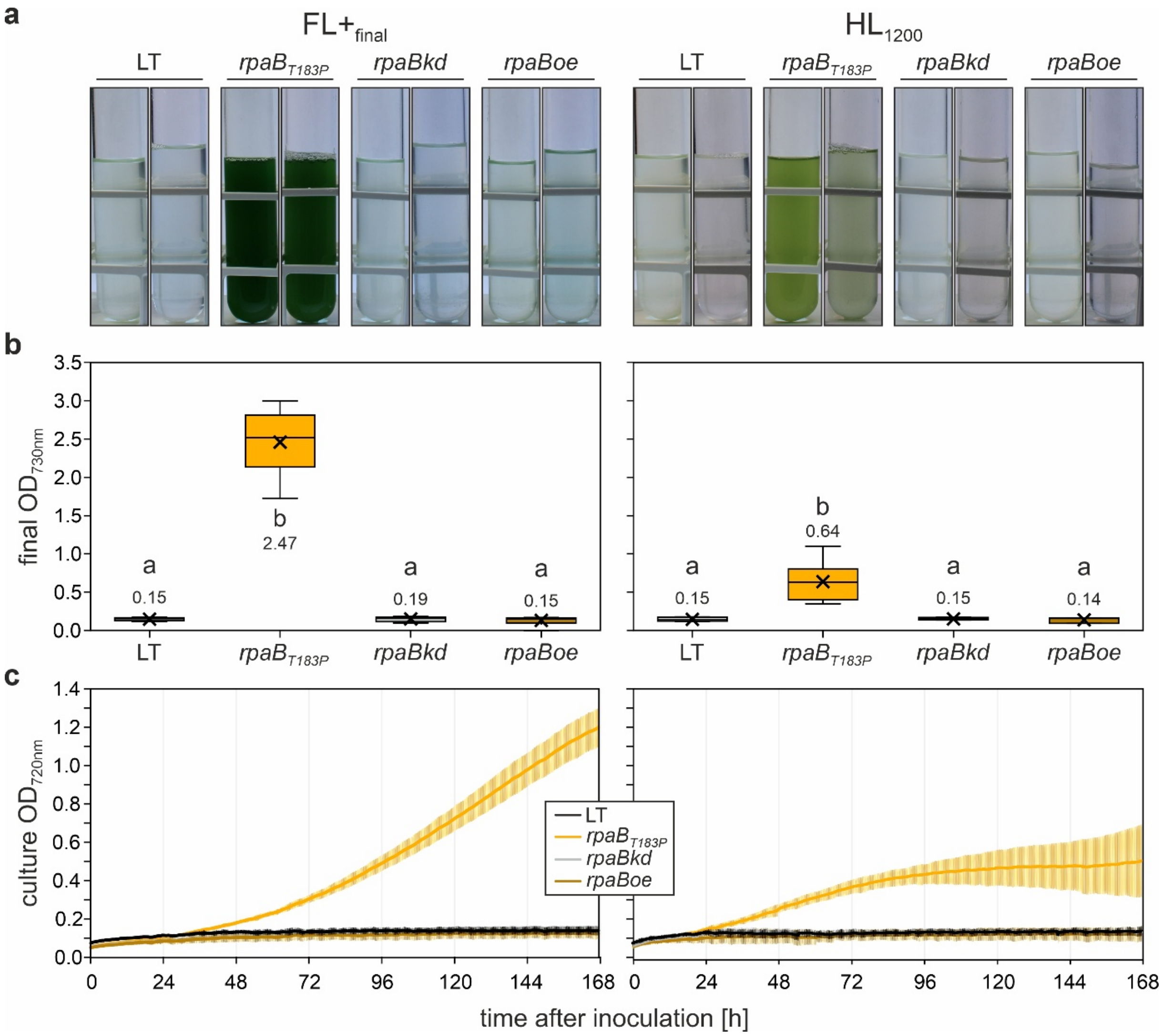

Supplementary Fig. 7 l Growth characteristics of the *rpaBkd*, *rpaBoe*, *rpaB*$_{T183P}$, and the LT strain under FL+$_{\text{final}}$ and constant HL$_{1200}$ conditions.

a, Visual representation of liquid cultures of the four strains cultivated in multi-cultivators under FL+$_{\text{final}}$ and constant HL$_{1200}$ conditions at 23 °C with 100 mL min^{-1} aeration, photographed seven days post-inoculation.

b, Quantitative analysis of cell density (OD$_{730\text{nm}}$) for the four strains grown under conditions as in a. Box plots are presented, with lowercase letters denoting statistically significant differences ($p \leq 0.05$) as determined by post-hoc Bonferroni-Holm simultaneous comparison of all measurements following significant between-group differences detected by one-factorial ANOVA.

c, Growth kinetics of the four strains under final FL+$_{\text{final}}$ conditions, monitored automatically by multi-cultivators measuring OD$_{720\text{nm}}$. The solid lines show the mean, and error bars represent the standard deviation. Data shown in panels b and c represents n = 5/8/5/4 biological replicates of LT/*rpaB*$_{T183P}$/*rpaB*$_{kd}$/*rpaB*$_{oe}$, respectively. HL$_{1200}$ data shown in panels B and C represents n = 4/6/4/3 biological replicates of LT/*rpaB*$_{T183P}$/*rpaB*$_{kd}$/*rpaB*$_{oe}$, respectively.

Statistical data in panel b are presented as box plots, showing individual data points, median (horizontal lines), mean (crosses), interquartile range (box), and 1.5× interquartile range (whiskers). Raw data are provided in **Source Data 6.**

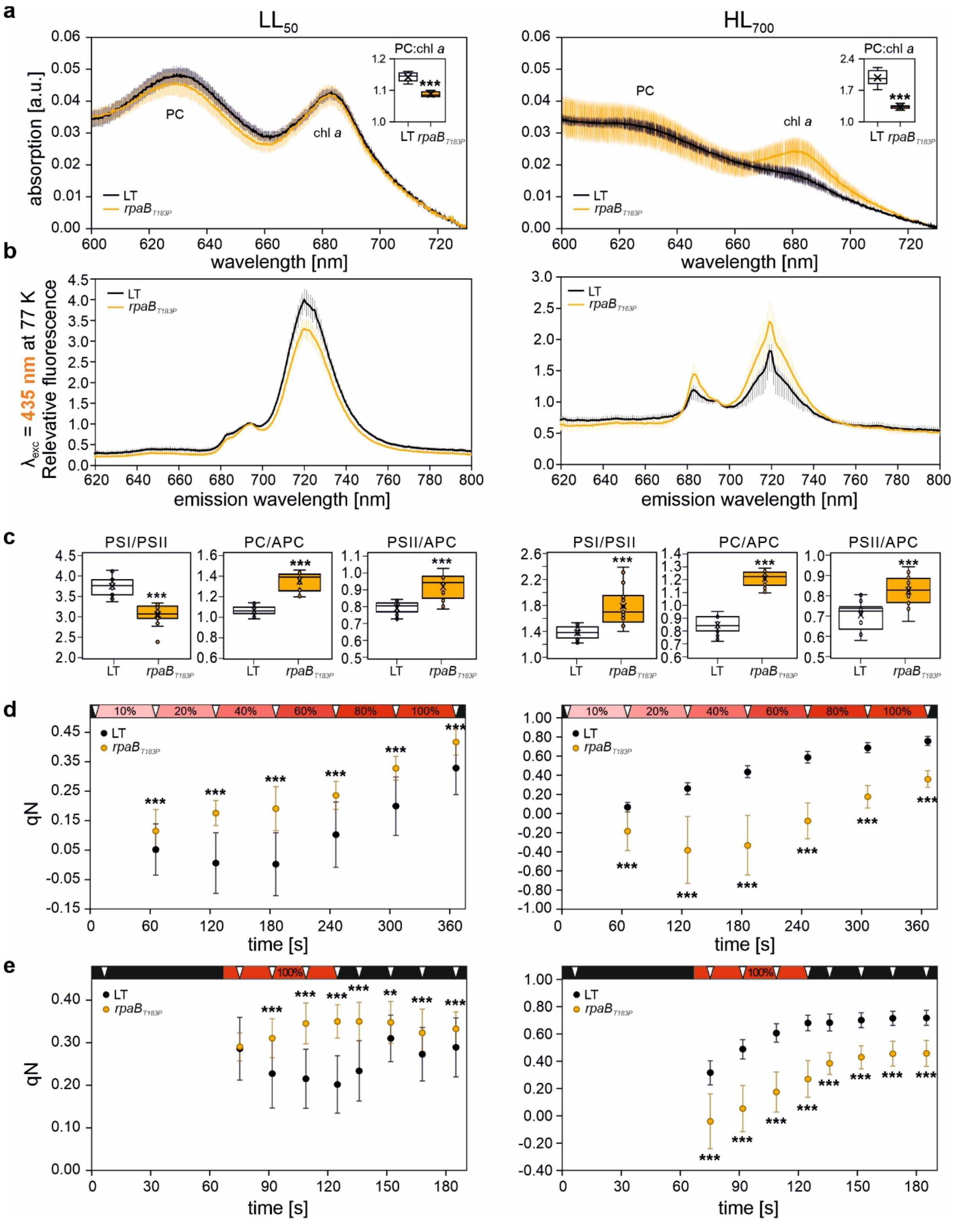

Supplementary Fig. 8 l Spectroscopic characterization of *$rpaB_{T183P}$*.

a, Absorption spectra. Absorption spectra of cultures grown under LL_{50} and HL_{700} were recorded at 0.2 nm intervals after dilution to OD_{730}=0.1. Data are shown as average ± SD. Absorption shoulders corresponding to phycocyanin (PC) and chlorophyll a (Chl a) are indicated. The inset box plot shows PC:Chl a ratios, calculated from absorption maxima (Abs_{630}:Abs_{684}). Biological replicates: n = 4 (LL_{50}) and n = 12 and 24 for LT and *$rpaB_{T183P}$*,

respectively (HL_{700}). Significant differences were observed ($p = 6.93\times10^{-4}$ for LL_{50}; 1.24×10^{-6} for HL_{700}).

b, Fluorescence emission spectra. Fluorescence emission spectra were measured at 77 K using deep blue excitation (435 nm, targeting Chl a) from undiluted samples (*n*=15 biological replicates for LL_{50}, *n*=16 biological replicates for HL_{700}). Spectra were recorded at 1 nm intervals, normalized to the PSII peak (F_{695}), and are presented as mean ± SD.

c, Photosystem and phycobilisome ratios. Box plots show PSI:PSII ratios derived from non-normalized fluorescence (F_{725}:F_{695}) from panel b. Additional ratios, PC:APC (F_{645}:F_{662}) and PSII:APC (F_{695}:F_{662}), were obtained from 77 K spectra using 600 nm excitation (see **Source Data 6**). Highly significant differences were observed under both conditions (LL_{50}: $p = 1.25\times10^{-8}$ for PSI/PSII, 3.96×10^{-14} for PC/APC, 2.56×10^{-7} for PSII/APC; HL_{700}: $p = 1.12\times10^{-5}$, 7.99×10^{-16}, and 5.13×10^{-5}, respectively).

d and **e,** Room temperature fluorescence and NPQ. Fluorescence was measured in dark-acclimated, OD_{730}-normalized cells under incremental red-orange illumination (**d**) and induction–relaxation (**e**) regimes. Illumination conditions included actinic light (~215 µmol photons m^{-2} s^{-1}, λ_{max} = 625 nm), dark periods, and saturating pulses (~1200 µmol photons m^{-2} s^{-1}). The non-photochemical quenching coefficient (qN) was calculated during and after illumination. Biological replicates: *n*=40 (LL_{50}); *n*=12 and 24 for LT and *rpaB*$_{T183P}$, respectively (HL_{700}).

Data in **a** and **c** are presented as box plots showing individual data points, median (horizontal lines), mean (crosses), interquartile range (box), and 1.5× interquartile range (whiskers).

Statistical significance was assessed using two-sided Student's t-tests (*$p \leq 0.05$, **$p \leq 0.01$, ***$p \leq 0.001$).

Raw data are provided in **Source Data 6**.

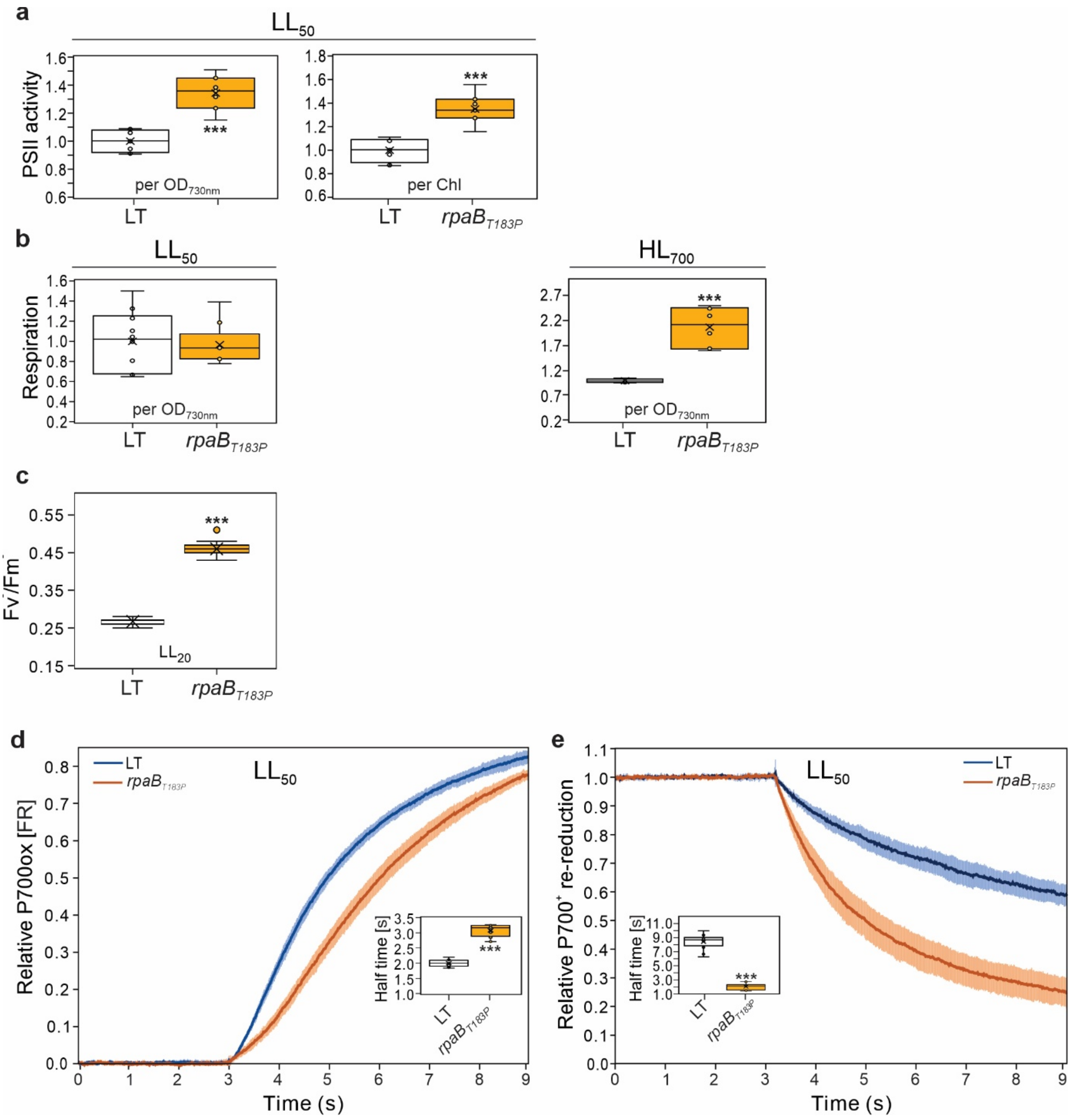

Supplementary Fig. 9 l Physiological measurements of *rpaB$_{T183P}$*.

a, PSII activity of the cells was obtained as described in **Supplementary Fig. 6a.** Measurements were performed on undiluted samples grown for 7 days at LL_{50} (n = 8 biological replicates for LT, and n = 7 for *rpaB$_{T183P}$*). The data were normalized to OD_{730nm} =1 and Chl a content of the cultures. The mean activities taken for LT and *rpaB$_{T183P}$* cells were 0.961 ± 0.071, and 1.290 ± 0.116 µmol O_2/mL/h, and 205 ± 21, and 278 ± 31 µmol O_2/mg Chl/h, respectively. p=1.54x10^{-5} and 3.68x10^{-5}.

b, Cellular respiration rates were obtained as described in **Supplementary Fig. 6b** (n = 10 biological replicates for LL_{50}, and n = 6 for HL_{700}). The mean activities taken for LT and *rpaB$_{T183P}$* cells were 0.084 ± 0.034, and 0.076 ± 0.016 µmol O_2/mL/h under LL_{50}, and 0.141 ±

0.032 and 0.264 ± 0.023 µmol O_2/mL/h under HL_{700}, respectively. p=$7.76x10^{-1}$ for LL50, and $6.31x10^{-5}$ for HL_{700}, respectively.

c, Room temperature fluorescence was measured as described in **Supplementary Fig. 6c** (*n* = 22 biological replicates). p=$2.45x10^{-38}$.

d and **e**, P700 oxidation (**d**) and re-reduction (**e**) kinetics were determined as described in **Supplementary Fig. 6d** and **e**. Twelve biological replicates were used for LT, and 7 for $rpaB_{T183P}$. p=$2.33x10^{-13}$ in (**d**) and $4.40x10^{-15}$ in (**e**).

Statistical data are presented as box plots, showing individual data points, median (horizontal lines), mean (crosses), interquartile range (box), and 1.5× interquartile range (whiskers). Statistical significance was determined using two-sided Student's t-Test, with *$p \leq 0.05$, **$p \leq 0.01$, and ***$p \leq 0.001$.

Raw data are provided in **Source Data 6**.
