# Supplementary material for: Improving tolerance to fluctuating light through adaptive laboratory evolution in the cyanobacterium Synechocystis
Source: Nat Commun. 2026 May 4;17:4025. doi: 10.1038/s41467-026-72689-x (PMC13139465; doi:10.1038/s41467-026-72689-x)
Supplement: Supplementary file 2 — Reporting Summary [file 41467_2026_72689_MOESM2_ESM.pdf]

nature portfolio

Corresponding author(s): Dario Leister

Last updated by author(s): Apr 9, 2026

Reporting Summary

Nature Portfolio wishes to improve the reproducibility of the work that we publish. This form provides structure for consistency and transparency in reporting. For further information on Nature Portfolio policies, see our Editorial Policies and the Editorial Policy Checklist.

Statistics

For all statistical analyses, confirm that the following items are present in the figure legend, table legend, main text, or Methods section.

n/a	Confirmed	
☐	☒	The exact sample size (*n*) for each experimental group/condition, given as a discrete number and unit of measurement
☐	☒	A statement on whether measurements were taken from distinct samples or whether the same sample was measured repeatedly
☐	☒	The statistical test(s) used AND whether they are one- or two-sided<br>*Only common tests should be described solely by name; describe more complex techniques in the Methods section.*
☒	☐	A description of all covariates tested
☐	☒	A description of any assumptions or corrections, such as tests of normality and adjustment for multiple comparisons
☐	☒	A full description of the statistical parameters including central tendency (e.g. means) or other basic estimates (e.g. regression coefficient) AND variation (e.g. standard deviation) or associated estimates of uncertainty (e.g. confidence intervals)
☐	☒	For null hypothesis testing, the test statistic (e.g. *F*, *t*, *r*) with confidence intervals, effect sizes, degrees of freedom and *P* value noted<br>*Give P values as exact values whenever suitable.*
☒	☐	For Bayesian analysis, information on the choice of priors and Markov chain Monte Carlo settings
☒	☐	For hierarchical and complex designs, identification of the appropriate level for tests and full reporting of outcomes
☒	☐	Estimates of effect sizes (e.g. Cohen's *d*, Pearson's *r*), indicating how they were calculated

Our web collection on statistics for biologists contains articles on many of the points above.

Software and code

Policy information about availability of computer code

Data collection	Chlorophyll fluorescence data was collected using FluorCam 7 Software (Photon Systems Instruments, Drasov, Czech Republic) and DualPAM software version 3.20 (Waltz, Effeltrich, Germany)<br>77K fluorescence spectra were collected using FluorEssence™ Software (HORIBA Scientific, Oberursel, Germany)<br>Growth curve data was collected using OD View Software (Photon Systems Instruments, Drasov, Czech Republic)<br>Enhanced chemiluminescence data was collected using FUSION FX software (Vilber, Collégien, France)<br>P700 redox kinetics data was collected using DualPAM software version 3.20 (Waltz, Effeltrich, Germany)
Data analysis	the quality of the WGS raw data was assessed using FastQC v0.11.9<br>pre-processing was performed using Cutadapt v4.1<br>k-mer coorection was performed using Rcorrector<br>mutation detection was performed using the Breseq pipeline<br>clean reads were aligned to the Synechocystis sp. PCC 6803 reference genome (ASM972v1) using bowtie2 v2.5.1<br>polymorphic and fixed mutations were identified by Breseq<br>phylogenetic analyses were performed using IQ-TREE multicore version 2.2.6<br>phylogenetic tree generation was performed using CLC Main Workbench (QIAGEN, Venlo, Netherlands)<br>protein 3D structure prediction based on amino-acid sequenced was performed using AlphaFold3 (https://alphafoldserver.com/)

For manuscripts utilizing custom algorithms or software that are central to the research but not yet described in published literature, software must be made available to editors and reviewers. We strongly encourage code deposition in a community repository (e.g. GitHub). See the Nature Portfolio guidelines for submitting code & software for further information.

Data

Policy information about availability of data

All manuscripts must include a data availability statement. This statement should provide the following information, where applicable:

- Accession codes, unique identifiers, or web links for publicly available datasets
- A description of any restrictions on data availability
- For clinical datasets or third party data, please ensure that the statement adheres to our policy

All source data of this study is provided in the supplementary data sets. Biological material is available upon reasonable request.
DNA-Seq and RNA-Seq data are available from the NCBI SRA database under Project number: PRJNA1228058 and Project number: PRJNA1372014.

Research involving human participants, their data, or biological material

Policy information about studies with human participants or human data. See also policy information about sex, gender (identity/presentation), and sexual orientation and race, ethnicity and racism.

Reporting on sex and gender	not applicable
Reporting on race, ethnicity, or other socially relevant groupings	not applicable
Population characteristics	not applicable
Recruitment	not applicable
Ethics oversight	not applicable

Note that full information on the approval of the study protocol must also be provided in the manuscript.

Field-specific reporting

Please select the one below that is the best fit for your research. If you are not sure, read the appropriate sections before making your selection.

☒ Life sciences ☐ Behavioural & social sciences ☐ Ecological, evolutionary & environmental sciences

For a reference copy of the document with all sections, see nature.com/documents/nr-reporting-summary-flat.pdf

Life sciences study design

All studies must disclose on these points even when the disclosure is negative.

Sample size	wherever possible, at least three biological replicates were assayed. The sole exception are evolving batch cultures, for whose characterization only one biological replicate could be available at any given time. For each characterized batch culture, quadruplicates inoculated from the same batch were investigated.
Data exclusions	no data was excluded from the analyses
Replication	Experimental findings were replicated at least trice (n>=3). All data presented could be reproduced within the range determined through given average values and stadard deviations.
Randomization	not applicable
Blinding	not applicable

Reporting for specific materials, systems and methods

We require information from authors about some types of materials, experimental systems and methods used in many studies. Here, indicate whether each material, system or method listed is relevant to your study. If you are not sure if a list item applies to your research, read the appropriate section before selecting a response.

Materials & experimental systems

n/a	Involved in the study	
☐	☒	Antibodies
☒	☐	Eukaryotic cell lines
☒	☐	Palaeontology and archaeology
☒	☐	Animals and other organisms
☒	☐	Clinical data
☒	☐	Dual use research of concern
☒	☐	Plants

Methods

n/a	Involved in the study	
☒	☐	ChIP-seq
☒	☐	Flow cytometry
☒	☐	MRI-based neuroimaging

Antibodies

Antibodies used	antibodies used in this study were obtained from Agrisera (anti-AtpsaA: AS06 172; anti-AtpsbA: AS05 084; anti-AtpsbB: AS04 038; anti-AtpsbC: AS11 1787; anti-AtpB: AS05 085) (Agrisera, Vännäs, Sweden)<br>anti-RpaB (Slr0947) antibody was obtained from PhytoAB (PHY5200A), San Jose, California, United States of America<br>For the detection of PAM68, anti-serum was provided by Prof. Dr. Jörg Nickelsen (LMU Munich, Germany)
Validation	Agrisera and PhytoAB antibodies as per their respective datasheets<br>anti-PAM68 as per Armbruster et al., 2010 (THE PLANT CELL)

Plants

Seed stocks	not applicable
Novel plant genotypes	not applicable
Authentication	not applicable
